# Developing a Feeding Module with a Blend of Garlic Oil and Cinnamon Bark for Enhancing Antioxidant Status and Immunity of Murrah Buffalo (*Bubalus bubalis*) with an Improvement in Feed Efficiency and Reduced Methane Emissions

**DOI:** 10.3390/antiox14060702

**Published:** 2025-06-10

**Authors:** Avijit Dey, Shubham Thakur, Ram Kumar Singh, Sandeep Sheoran, Jerome Andonissamy, Sanjay Kumar

**Affiliations:** 1 Division of Animal Nutrition and Feed Technology, ICAR-Central Institute for Research on Buffaloes, Hisar 125 001, Haryana, India; 2 Division of Animal Physiology and Reproduction, ICAR-Central Institute for Research on Buffaloes, Hisar 125 001, Haryana, India; 3 Division of Animal Genetics and Breeding, ICAR-Central Institute for Research on Buffaloes, Hisar 125 001, Haryana, India

**Keywords:** garlic oil, cinnamon bark, feeding module, immune response, antioxidant status, growth, methane emissions, buffalo

## Abstract

The experiment was designed to evaluate the consequence of a blend of garlic oil and cinnamon bark powder administration on growth performance, nutrient digestibility, immunity, antioxidant status and methane emission in Murrah buffalo (*Bubalus bubalis*). Sixteen buffalo calves were divided into two groups in a completely randomised design. The first group (CONT) was fed a basal diet of wheat straw, green oats and concentrate mixture, whereas the second group (GOCB) received feeds as per the CONT along with a blend of garlic oil and cinnamon bark powder (0.5 mL + 1.0 g/head/day) by mixing it with the concentrate mixture for a period of 170 days. The growth rate and feed efficiency in GOCB group buffalo calves were improved (20%) with better (*p* < 0.05) digestibility of organic matter and crude proteins. Buffaloes of the GOCB group revealed enhanced (*p* < 0.05) immunity and antioxidant enzymes with reduced (*p* < 0.05) lipid peroxidation (26% less MDA production). The methane concentration in the eructed gas of the GOCB buffaloes was reduced (33.88%) in comparison with the CONT (*p* < 0.01). Thus, feed formulated with a blend of garlic oil-cinnamon bark powder demonstrates improvements in the health and production performances of buffalo calves.

## 1. Introduction

Nutrition plays a crucial role in the prevention and control of various diseases through the enhanced antioxidant status and immune response of animals, besides improvement in production and reproduction performances [1,2,3]. Diet can modulate host immunity by several mechanisms viz. direct stimulation of the cells of the immune system, the release of enzymes and hormones as well as improved utilisation of nutrients through modulation of the gut microbiome [4,5]. In ruminants, the gut microbiome plays a major role in the digestion and assimilation of feeds through ruminal fermentation by a complex microbial system. Methane is the byproduct of rumen fermentation, produced by methanogenic archaea during the fermentation of feeds in the rumen, and is an essential process, which represents a loss of about 8–10 percent of the gross energy of feed [6].

The rapid degradation of dietary proteins by ruminal proteolytic bacteria and hyper-ammonia-producing bacteria (HAB) results in excess ammonia production, beyond the capacity of rumen microbes for the synthesis of microbial protein [7]. As protein is the costliest ingredient in ruminants’ rations, there is economic loss for livestock owners due to the non-utilisation of dietary protein for productive purposes, besides environmental pollution through the excretion of N via faeces and urine. Therefore, ruminant nutritionists are in search of dietary modulation for enhancing fibre digestion with a concomitant reduction in ruminal protein degradation and methanogenesis. Modulation of diet through the addition of essential oil-rich feed supplements has been demonstrated to enhance nutrient utilisation and reduce methanogenesis and ruminal proteolysis; however, their effectiveness varies on dose levels and types of essential oils [8,9]. In addition, bioactive compounds present in essential oils have been reported to enhance antioxidant status and immunity in a few sporadic studies conducted in livestock and poultry [10,11]. As these essential oil compounds have strong antibacterial activities, which may negatively impact the rumen fermentation [12], a blend of essential oil compounds at low dose levels could synergistically interact with each other to modulate rumen fermentation for better nutrient utilisation, reduced methanogenesis as well as improved antioxidant status and immunity of animals.

Buffalo husbandry plays a pivotal role in the development of the rural economy of many Asian countries. With 109.8 million heads of buffalo population, India is the leader in buffalo-centric development by having the world’s highest milk production and significantly contributing to buffalo meat (carabeef) production. Out of 21 recognised breeds in the country, 42% of the Indian buffalo population is the world-famous Murrah, which is recognised as the ‘Black Gold’ of the country [13]. Buffalo is considered a good converter of fibrous feeds to animal products and therefore has a large scope for sustainable food production in the era of climate change [14]. However, a significant quantity of greenhouse gases in the country is contributed by buffalo by the enteric methane emissions and manure [15]. Therefore, there is an urgent need to develop a sustainable feeding module for enhancing antioxidant status and immunity to prevent the occurrence of diseases as well as enhance production performances with reduced greenhouse gas emissions.

Garlic oil (*Allium sativum*) is rich in organosulphur compounds, allicin, diallyl sulphide and diallyl disulphide, which has been demonstrated to have strong antimicrobial activity to alter rumen fermentation in a dose-dependent manner [16]. High doses have been reported to negatively impact feed digestion and volatile fatty acids production. Garlic oil could also reduce methanogenesis by inhibiting ruminal archaea through the inhibition of the 3-hydroxy-3-methylglutaryl coenzyme A (HMG CoA) reductase enzyme, necessary for archaeal cell wall synthesis [17]. Cinnamon bark contains cinnamaldehyde, a major bioactive compound, which is antibacterial, antiparasitic and anti-inflammatory, and is demonstrated to modulate rumen fermentation by altering ruminal bacterial and archaeal populations [18,19]. However, to the best of our knowledge, very limited studies have been conducted on the application of these compounds either individually or in blends for improving the health and production performances of buffaloes. In a study, Oh et al. [20] reported the immuno-stimulatory effects of various phytonutrients (garlic, capsicum and curcumin) without affecting the antioxidant status of dairy cows. In contrast, Imbadi et al. [21] demonstrated an enhanced immunity and antioxidant status in goats supplemented with garlic and other essential oils. Many researchers demonstrated enhanced antioxidant status and production performances in dietary supplementation of cinnamaldehyde in pigs, broilers and dairy cows; however, Chaves et al. [22] reported no effects on the growth rate, rumen fermentation and meat quality of lambs supplemented with cinnamaldehyde in concentrate-based diets. The differences in the activity of various garlic products and other essential oils could be attributed to physiological status, animal species and the concentrations, forms and composition of bioactive compounds fed to the animals. The present study, therefore, was planned to develop a feeding module with a blend of garlic oil and cinnamon bark powder and to investigate its effects on antioxidant status, immunity and blood biochemical indices along with growth rate, nutrient utilisation and methane emission in buffalo (*Bubalus bubalis*) calves.

## 2. Materials and Methods

The present feeding experiment was conducted at the Animal Nutrition Research Shed no. 9 of the animal farm section, ICAR—Central Institute for Research on Buffaloes, Hisar, India (29.18618° N, 75.70083° E). All of the experimental procedure was carried out with prior approval of the Institute Animal Ethics Committee (IAEC) for the care and management of animals (IAEC-CIRB/20–21/05, dated 10 June 2020).

### 2.1. Collection and Preparation of Blend of Garlic Oil and Cinnamon Bark

The garlic (*Allium sativum*) oil (W250309, CAS no. 8000-78-0) was purchased from Sigma-Aldrich Ltd. (Bengaluru, India), and the cinnamon (*Cinnamomum verum*) bark was purchased from a local market, Hisar, Haryana, India. The cinnamon bark was dried overnight at 60 °C in a hot air oven and ground in a mixer grinder to create fine powder. A blend of garlic oil (0.5 mL) and cinnamon bark powder (1.0 g) was prepared and kept airtight for further use in animal studies.

### 2.2. Experimental Animals, Diet and Management

**Figure 1:**
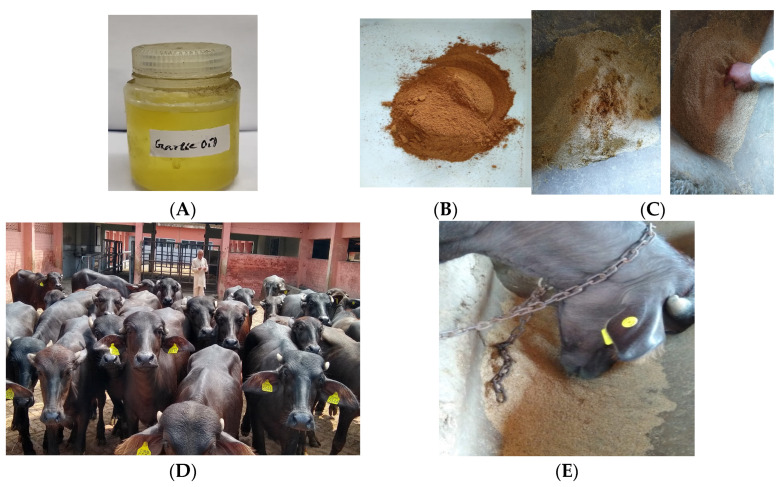
Preparation and feeding of blend of garlic oil and cinnamon bark powder to buffalo calves. (**A**): Garlic oil; (**B**): cinnamon bark powder; (**C**): blend of garlic oil-cinnamon bark powder (0.5 mL + 1.0 g) premixed with a small quantity of concentrate feeds; (**D**): buffalo calves for experiment; (**E**): mixing of the premix with the total required concentrate feeds and placed in the manger for feeding to buffalo calves.

Sixteen Murrah buffalo (*Bubalus bubalis*) female calves 8–12 months old with an average body weight of 183.87 kg were obtained from the animal farm section of the institute and randomly divided into two groups of eight animals in a completely randomised design. The animals were kept in a house with proper ventilation and open-paddock arrangement for movements. The shed was equipped with mangers for monitoring the feeding of individual animal. Fresh drinking water was provided to all the animals thrice daily throughout the experimental feeding. The control group animals (CONT) received a control diet containing ad lib wheat straw, fresh oats green (5 kg) and a required quantity of concentrate feed mixture (Table 1) to support the nutritional requirements of protein and energy for the maintenance and growth of the animals [14], whereas the treatment group (GOCB) received the control diet along with the blend of garlic oil-cinnamon bark [(0.5 mL + 1.0 g)/ head/day)], which was premixed with a small portion of concentrate mixture (Figure 1) before mixing it to the requisite quantity. The dose level of garlic oil-cinnamon bark for feeding to the buffalo calves was determined on the findings of a series of in vitro rumen fermentation studies conducted at authors’ laboratory [16,23,24]. This dose was applied for calculating the dose in growing calves of 180 kg body weight assuming a rumen volume of 10% of body weight and 20% higher than the calculated dose to account for calculation errors and a dynamic system. Based on these calculations, buffalo calves were fed with a blend of 0.5 mL of garlic oil and 1.0 g of cinnamon bark powder daily mixed with concentrate mixture. For homogeneous distribution, the blend was premixed manually with a small quantity of concentrate feeds; then, the premix was mixed with the total required quantity of concentrate feeds and fed to the animal individually. The feeding experiment was conducted for 190 days including 20 days of adaptation to new feeds and 170 days of concrete data recording and sample collection for analysis. The additive mixed-concentrate feed was added with a gradual increase to the control concentrate mixture for suitable adaptation to the new feeding regimen and to achieve the desired dose level within 10 days.

### 2.3. Recording of Feed Intake and Body Weight Changes

The daily feed intake of all the experimental animals was recorded individually at every fortnight by precisely recording offered feeds and leftovers after 24 h. The growth rate of buffalo calves was determined by recording the fortnightly body weight of the individual animal before offering feed and water by a digital weighing balance throughout the study of 170 days. The feed conversion ratio (FCR) and feed efficiency (FE%) of buffalo calves were calculated by considering daily feed dry matter intake and body weight gain to determine the feeding value of concentrate feed mixture containing blended garlic oil and cinnamon bark powder.

### 2.4. Haemato-Biochemical Studies

About 10 mL of blood sample was collected from each animal through jugular vein puncture before morning feeding at 0, 90 and 170 days of the experimental feeding in two vacutainers, one with anticoagulant EDTA and the other without any anticoagulant. Haemoglobin (Hb) and packed cell volume (PCV) was estimated from whole blood collected with anticoagulant. Blood collected without anticoagulant was kept undisturbed for 30 min, and serum was collected from the supernatant and put in a −20 °C deep freezer for serum chemistry analysis. Total protein, albumin, alanine amino transferase (ALT), aspartate amino transferase (AST) and serum urea concentrations were analysed by biochemical assay kits (Coral Clinical Systems, Goa, India). The globulin concentration was determined by subtracting albumin concentration from total protein, and the ratio of albumin to globulin (A: G) was calculated.

### 2.5. Erythrocytic Antioxidant Status

The total thiol (T-SH) groups and antioxidant enzymes viz. reduced glutathione (GSH), catalase (CAT), superoxide dismutase (SOD) and lipid peroxidase (LPO) in the erythrocytes were estimated to study the effects of garlic oil-cinnamon bark-supplemented feed in buffalo calves. After the collection of blood in a heparinised vacutainer from the jugular vein of all calves, the blood was centrifuged at 1800× *g* for 15 min. The plasma and buffy coat were decanted carefully and washed with normal saline solution thrice, and the erythrocyte pellet was collected. A uniform RBC suspension, i.e., haemolysate, was prepared by mixing (1:1) the packed RBC in the erythrocyte pellet with ice-cooled distilled water. A working haemolysate was prepared by mixing 1 mL of RBC suspension with 9 mL of distilled water. The prepared haemolysate was stored in a deep freezer (−20 °C) for analysing erythrocytic antioxidant status. The total thiol (T-SH) group in haemolysate was analysed as per the procedure of Sedlak and Lindsay [25]. The molar extinction coefficient of 13100 at 412 nm was used to estimate the thiol contents, and the values were expressed in µmol per mg of haemoglobin. The dithio-bis-2 nitro benzoic acid (DTNB) method [26] was adopted to estimate GSH, where sodium tungstate solution was added to haemolysate and centrifuged, and optical density was measured at 412 nm, spectrophotometrically after adding Tris buffer and DTNB reagent to supernatant fluid. The CAT activity in the haemolysate was estimated [27] by using hydrogen peroxide solution and measuring optical density at 230 nm at various time points. The MTT (3-[4-5 dimethyl thiazol 2-xl] 2, 5 diphenyl tetrazolium bromide) reduction was analysed to determine the SOD activity [28], where mmol MTT formazon formed per mg haemoglobin was measured. The quantity of malonyl dialdehyde (MDA), a product of lipid peroxidation (LPO), was estimated [29] to examine LPO level. The haemoglobin (Hb) concentration in the haemolysate was estimated by a colorimetric assay [30] with 0.007 N ammonium hydroxide solution, and the antioxidant enzyme activities were demonstrated.

### 2.6. Immunity Assessment

The immunity status of buffalo calves on feeding of a garlic oil-cinnamon bark-based diet were evaluated by investigating cell-mediated immune response (CMI) and humoral immune (HI) response [31]. A delayed type of hypersensitivity reaction caused by injection of phytohaemagglutinin-P (PHA-P) (Sigma-Aldrich Ltd., New Delhi, India, CAS No. 9008-97-3) to buffalo calves was assessed for evaluating CMI response. After proper marking of both sides of the neck region, PHA-P (150 µg/200 μL PBS solution) was injected intra-dermally. A digital vernier calliper was used to measure the increased skin thickness due to the hypersensitivity reaction at 0, 24, 48, 72 and 96 h post-injection.

The buffalo calves were vaccinated against haemorrhagic septicaemia (HS), and the antibody titre was measured to demonstrate the HI response. *Pasteurella multocida* culture, inactivated with formaldehyde, in potassium aluminium sulphate adjuvant was obtained from Haryana Veterinary Vaccine Institute (Department of Animal Husbandry and Dairying, Govt. of Haryana, India). After experimental feeding of 60 days, a single subcutaneous dose (5 mL) of HS vaccine was injected to each buffalo calf, and antibody titre was measured in plasma after collection of blood from the jugular vein in a heparinised vacutainer at 0, 30 and 60 days post-vaccination. The antibody titre (log10) was measured by recording optical density at a 450 nm wavelength by an Enzyme Linked Immuno-Sorbent Assay (ELISA) examination at Lala Lajpat Rai University of Veterinary and Animal Sciences (LUVAS), Hisar 125 004, Haryana, India.

### 2.7. Digestion Trial and Sampling Protocol

After 90 days of feeding trial, a digestion trial of 8 days duration was conducted, where 2 days of adaptation of the animals to the experimental condition was considered prior to 6 days total of collection. The daily feed intake was determined by recording the feeds offered and measuring refusal. The daily total faeces voided by each buffalo calf were collected and weighed. After thorough mixing of the faeces individually, a sample (1/200th of total faeces) was dried for 24 h in a forced hot air oven (NSW, New Delhi, India) set at temperature of 80 ± 2 °C. The faecal dry matter was determined, and the 6 days animal-wise collection was mixed together and kept for chemical analysis after grinding to a 1 mm particle size. A portion (1/400th of total faeces) of fresh faeces sample from individual calves was collected daily and kept in plastic bottles added with 10 mL of 25% (*v*/*v*) sulphuric acid. The 6 days of collection in each bottle was mixed, and faecal N was analysed. The digestibility of nutrients was calculated considering the intake and faecal output of each nutrient for individual animals.

### 2.8. Enteric Methane Production Measurement

Towards the end of feeding trial (90 days onwards for 3 consecutive days), the eructed gas from all the experimental animals was collected at 3 h post-feeding through the Douglas bag technique [8], and methane concentration was analysed by a gas chromatograph (Nucon-5700, Nucon Engineers, New Delhi, India). The GC was equipped with a flame ionisation detector (FID) and Porapak-Q (length-1.5; mesh range 80–100; o.d. 3.2 mm) packed stainless steel column. The oven, injector and detector temperatures were set to 40 °C, 50 °C and 50 °C, respectively. A methane and CO_2_ mixed (50:50) gas standard (Centurion Scientific, New Delhi, India) was used for comparison. The main and column pressure for carrier gas N_2_, column pressure for fuel H_2_ and zero-moisture air was set to 30, 10, 20 and 10 PSI, respectively. The concentration of methane (%) was calculated on measuring the peak area covered by the sample and standard.

### 2.9. Chemical Analyses

The feeds, refusals and faeces samples collected during the digestion trial were analysed as per the Association of Official Analytical Chemists [32] for DM (934.01), organic matter (967.05), crude protein (976.05), ether extract (973.18) and total ash (942.05). Neutral detergent fibre (NDF) and acid detergent fibre (ADF) were analysed without sodium sulphite and α-amylase [33].

### 2.10. Statistical Analyses

The experimental data (eight replicates in each treatment) were analysed for the various parameters as a completely randomised design using an independent sample *t*-test of SPSS v.20 [34]. The data were tested for normal distribution by the Shapiro–Wilk test, and repeated measures ANOVA tests were used for the data recorded at different time points. The difference between the two-treatment means was determined by independent samples *t*-tests in SPSS as per Snedecor and Cochran [35], and significant variations were demonstrated when *p* ≤ 0.05.

## 3. Results

The chemical composition of feeds fed to buffalo calves, comprising concentrate mixture, oats green fodder and wheat straw (Table 1), supports the nutrient requirement of buffalo calves for maintenance and growth [14].

Although all the experimental buffalo calves [control (CONT) and garlic oil-cinnamon bark blend (GOCB)] had comparable (*p* > 0.05) body weights at the start and completion of trial, the total body weight gain after 170 days of experimental feeding and average daily gain remained higher (*p* < 0.05) in GOCB in comparison with CONT (Table 2). The quantities of wheat straw, oats green and concentrate mixtures with/without GOCB given to each animal of either group during the entire experimental feeding period of 170 days remained similar, resulting in comparable (*p* > 0.05) total feed intake (kg/d) between the groups. As daily body weight gain was higher (*p* < 0.05) in GOCB animals, the lowered (*p* < 0.05) feed conversion ratio (FCR) indicating higher (*p* < 0.05) feed efficiency (FE) were demonstrated, owing to similar intake of feeds.

The haemoglobin (Hb), packed cell volume (PCV), serum proteins (albumin, globulin and A:G ratio) and serum enzymes (ALT and AST) levels remained comparable (*p* > 0.05) in buffalo calves, irrespective of feeding regimens at any time point of blood collected. However, a reduction (*p* < 0.05) in the serum urea level was demonstrated at 170 days in GOCB blend fed animals in comparison to control (Table 3).

The erythrocytic antioxidant indices measured by total thiol groups (T-SH), reduced glutathione (GSH), catalase (CAT) and superoxide dismutase (SOD) enzymes activities were enhanced (*p* < 0.01) in GOCB-fed calves in comparison with CONT; however, the lipid peroxidation (LPO) was reduced (*p* < 0.01) in GOCB, indicating better antioxidant status (Table 4).

All the buffalo calves showed the highest skin thickness (%) after 48 h injection of PHA-P, subsided gradually and came close to normal after 96 h (Table 5). At any post-injection time points (24, 48, 72 and 96 h), it remained enhanced (*p* < 0.05) for GOCB blend-supplemented calves compared with CONT, suggesting better immune response. The humoral immune response, depicted by plasma antibody titre (log10), remained higher (*p* < 0.05) both at 30 and 60 days after vaccination against *Pasteurella multocida* in GOCB blend-supplemented buffalo calves than CONT (Table 6).

The digestion trial data (Table 7) revealed enhanced (*p* < 0.05) digestibility of dry matter (DM) and organic matter (OM) in GOCB animals; however, the digestibility of crude fat (EE), neutral detergent fibre (NDF) and acid detergent fibre (ADF) remained comparable (*p* > 0.05) between GOCB and CONT buffalo calves. Compared with buffalo calves of the control group, the crude protein (CP) digestibility was enhanced (*p* < 0.01) in GOCB animals (Table 7).

The methane emission (conc. in eructed gas) was reduced (*p* < 0.01) in the GOCB animals fed a diet containing a blend of garlic oil and cinnamon bark powder (Table 8).

## 4. Discussion

**Figure 2:**
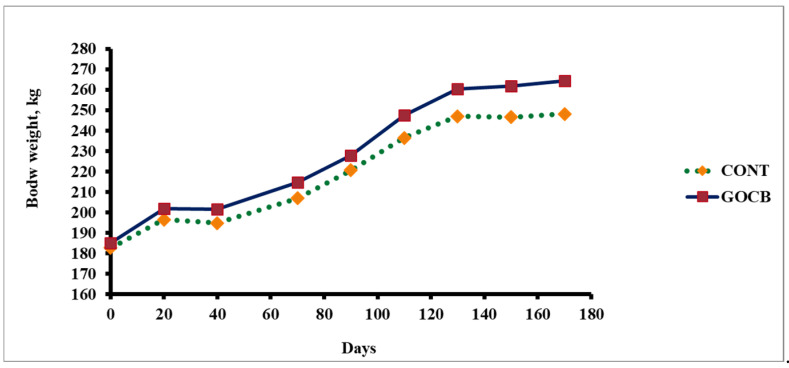
Body weight changes of buffalo calves (n = 8) fed a garlic oil-cinnamon bark blended feed mixture.

To the best of our knowledge, this is a unique study aiming to delineate the effects of a blend of garlic oil and cinnamon bark powder on body weight changes, feed utilisation, antioxidant enzymes and immunity in buffalo (*Bubalus bubalis*). Garlic (*Allium sativum*) oil contains several bioactive components viz. diallyl sulphide, allicin and allyl mercaptan [36,37], which have been reported to have influence not only on the ruminal microbial ecosystem but also on antioxidative and immune systems [38,39]. Cinnamon bark powder contains cinnamaldehyde, a major essential oil compound, which has the potential to act as an antimicrobial agent to modulate several functions of body [22,40]. The similar feed intake with enhanced body weight gain and feed efficiency in buffalo calves fed garlic oil-cinnamon bark (GOCB)-supplemented feed (Figure 2) could be due to increased assimilation of nutrients [41] through modulation of the rumen microbial ecosystem [37,42] by associative effects of garlic oil and cinnamon bark powder. In a study with growing lamb, Chaves et al. [22] reported no changes in feed intake and body weight gain when cinnamaldehyde alone was supplemented in the diet at 400 mg/kg dry matter. In contrary, an improvement in growth performance was demonstrated [43] in pigs supplemented with basal diet containing cinnamaldehyde (80 mg/kg feed). However, Holstein calves fed a high-concentrate diet supplemented with cinnamon essential oils (5 g/d) reported no effects on feed intake or body weight gain. Damascus goats supplemented (2 mL/d) with garlic oil [21] were reported with enhance growth rate and feed efficiency without affecting intake.

The normalcy of haemato-biochemical indices in all the experimental buffalo calves irrespective of diet demonstrated the good health and wellbeing of animals; however, reduced serum urea concentration could be due to selective inhibition of ruminal hyper-ammonia-producing bacteria by essential oil-containing garlic oils and cinnamon bark [44,45], demonstrating better protein utilisation in the gastro-intestinal tract. Our study receives support from other researchers [22,43], where a dose-dependent variation in serum urea concentration was described without it affecting other blood metabolites.

Endogenous antioxidant enzymes viz. superoxide dismutase (SOD), catalase (CAT), glutathione (GSH) and malondialdehyde (MDA) are important and play a significant role in counteracting reactive oxygen species (ROS) and preventing oxidative damage to body cells [46]. The increased (*p* < 0.01) levels of these antioxidant enzymes and erythrocytic total thiol group (T-SH) and reduced (*p* < 0.01) lipid peroxidation, as determined by less malonaldehyde (MDA) formation in the present study, demonstrated the better antioxidant status of animals fed a GOCB diet (Table 4). Several studies with plant bioactive compounds containing polyphenol and essential oils have been reported to enhance the activity of antioxidant enzymes [47,48,49,50]. The findings of Imbabi et al. [21] on supplementation of garlic oil, thyme or a blend of garlic oil and thyme in goats’ diet corroborate our study in terms of an improved antioxidant status of buffalo calves. Supplementation of garlic products enhances antioxidant enzymes and reduced malonaldehyde in lambs and Holstein cows [51,52] by regulating binding of antioxidant response elements for the expression of antioxidant enzymes. A reduction in lipid peroxidation and enhanced antioxidant enzymes were reported in pigs fed a diet containing cinnamaldehyde [43].

**Figure 3:**
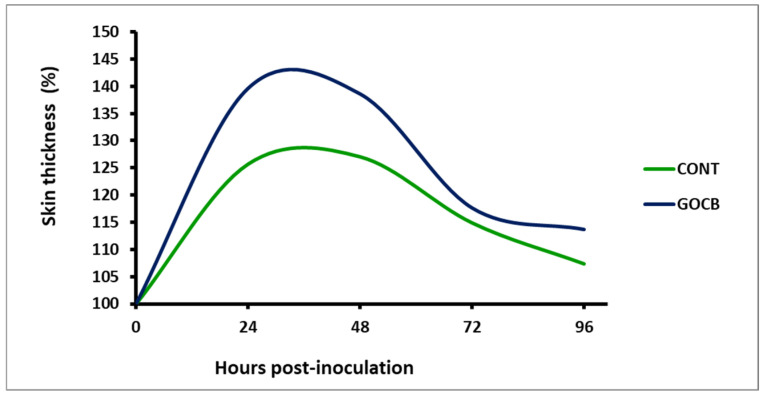
Skin fold thickness (%) of buffalo calves (n = 8) on intra-dermal administration of phyto-haemagglutinin-P.

**Figure 4:**
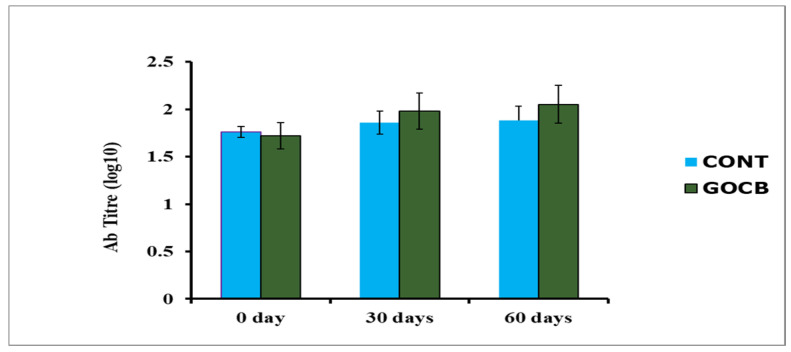
Antibody titre of buffalo calves (n = 8) against *Pasteurella multocida* vaccine.

Enhanced cell-mediated (Figure 3) and humoral immunity (Figure 4) in GOCB-fed animals in the present study could be due to enhanced immunoglobulin production through B-cell stimulation [53,54] or enhanced phagocytosis [55] or the reduction of stress through enhanced antioxidant enzymes (Table 4). Increased feed utilisation, especially proteins, as evidenced by the enhanced digestibility of the feed by GOCB animals (Table 7), also provides better immunity by supplying better nutrition [56] to the animals.

Improved immunity was also reported by Luo et al. [43] on supplementation of cinnamaldehyde to growing pigs. Similar to our study, goats supplemented with thyme and/garlic oil demonstrated higher immune status through improved growth and nutrient utilisation [21]. In another study, garlic powder supplementation to lambs was described to enhance immunoglobulin levels [53]. Overall, the improved feed utilisation, growth rate and antioxidant enzymes status of buffalo calves in the present study enhanced the immunity of GOCB-fed animals.

Essential oil-rich feed additives have been reported to modulate rumen fermentation by affecting ruminal microbes, which mostly depends on the type and dose of the bioactive compounds [57,58]. Ma et al. [59] reported decreased protozoa and increased cellulolytic bacterial population in the rumen of ewes supplemented with allicin, a bioactive compound of garlic oil. Improvements in digestibility and feed efficiency in growing lambs fed various essential oil compounds were demonstrated [60] through the stimulation of gastric enzymes and ruminal bacteria. The present study also corroborates with the findings of earlier researchers in enhancing the feed digestibility of GOCB-supplemented buffaloes (Table 7). As in the present study, an increased protein utilisation was demonstrated due to reduced ruminal ammonia N production [37,39], inhibition of ruminal hyper-ammonia-producing bacteria [45] and increased protein flow and utilisation in the small intestine [61,62].

**Figure 5:**
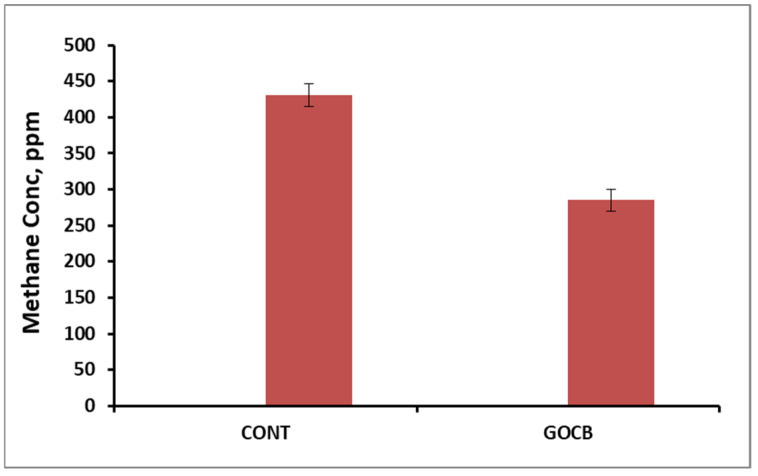
Methane concentration in the eructed air of control (n = 8) and garlic oil-cinnamon bark-supplemented animals (n = 8).

Bioactive plant compounds have been demonstrated to reduce enteric methane emissions due to their strong antimicrobial effect [63,64]. Direct reduction of methanogens through inhibition of archaeal membrane lipid synthesis [65] or reduction of ruminal protozoal population associated with methanogens [66] by essential oil compounds (thyme, carvacrol, cinnamaldehyde, garlic oil, etc.) could partially explain the reduced methane concentration (Figure 5) in the eructed air of GOCB-fed buffalo calves.

## 5. Conclusions

There is a great demand for feeds not only the enhancing growth and digestibility of nutrients but also enhancing the antioxidant status and immunity with a concomitant reduction of enteric methane emission. The present study delineated that the administration of a blend (1:2) of garlic oil and cinnamon bark powder (0.5 mL and 1.0 g/head/day) improved growth rate and feed efficiency (20%), protein utilisation (9.87%), antioxidant status (less MDA 26%) and immunity with reduction (33.88%) of methane emission in Murrah buffalo calves. Therefore, this study demonstrates the application of a garlic oil-cinnamon bark powder blend in the feed formulation of buffalo calves for improving health and production performances. There is also a need to carry out animal studies with varying dose levels at different feeding regimes to obtain the best possible result. Further studies investigating lactating animals should focus on the assessment of milk production and quality parameters.

## Figures and Tables

**Table 1:** Chemical compositions (% DM) of concentrate mixture, wheat straw and oats green fed to buffalo (*Bubalus bubalis*) calves.

Attributes	Concentrate Mixture ^1^ (n = 8)	Wheat Straw (n = 8)	Oats Green (n = 8)
Organic matter	93.16	92.17	92.62
Crude protein	20.22	4.24	7.48
Ether extract	5.35	1.31	2.27
Total ash	6.84	7.83	7.38
Neutral detergent fibre	39.53	81.08	64.31
Acid detergent fibre	10.12	52.80	37.50

^1^ Concentrate mixture consisted of wheat (25%); barley (10%); rice grit (5%); groundnut cake, expeller (26%); mustard cake, expeller (4%); wheat bran (27%); mineral mixture (2%); and common salt (1%).

**Table 2:** Feed intake, body weight gain and feed efficiency of buffalo calves fed different feeds.

Attributes	Treatments		SEM	*p* Value
	CONT (n = 8)	GOCB (n = 8)		
Body Weight (kg)				
Initial	182.70	185.04	12.90	0.935
Final	248.20	264.54	14.14	0.594
Total Gain ^δ^	65.50 ^a^	79.50 ^b^	3.07	0.01
ADG (g)	385.08 ^a^	467.66 ^b^	18.05	0.01
Dry Matter Intake (kg/d)				
Wheat Straw	1.61	1.73	0.184	0.763
Conc. Mix	1.82	1.82	0	1.00
Oats Green Fodder	1.16	1.16	0	1.00
Total	4.59	4.72	0.184	0.762
FCR	11.97 ^b^	10.11 ^a^	0.438	0.022
FE (%)	8.40 ^a^	10.08 ^b^	0.386	0.018

CONT (control group) fed diet containing wheat straw, concentrate mixture and green oats fodder; GOCB is the treatment group, fed a control diet supplemented with a blend of garlic oil and cinnamon bark powder @ (0.5 mL + 1.0 g)/head/day, respectively, by mixing it to a small proportion of concentrate mixture. ^δ^ After 170 days; ADG = average daily gain; FCR = feed conversion ratio; FE (%) = percent feed efficiency; ^a,b^ mean values bearing different superscripts within a row vary significantly (*p* < 0.05).

**Table 3:** Haemato-biochemical parameters of buffalo calves supplemented with diet containing garlic oil-cinnamon bark blend.

Parameters	Day	Treatments		SEM	*p* Value
		CONT (n = 8)	GOCB (n = 8)		
Hb (g/dL)	0	10.56	10.89	0.82	0.670
	90	10.92	11.05	0.77	0.751
	170	11.03	11.32	0.82	0.432
PCV (%)	0	35.62	35.472	1.48	0.547
	90	34.59	34.92	1.23	0.234
	170	35.64	34.79	1.85	0.502
Total Protein (g/dL)	0	7.86	7.52	0.29	0.162
	90	8.54	8.20	0.34	0.372
	170	8.20	8.34	0.41	0.421
Albumin (g/dL)	0	3.17	3.64	0.06	0.371
	90	3.23	3.15	0.03	0.431
	170	3.74	3.52	0.08	0.785
Globulin (g/dL)	0	4.69	3.88	0.43	0.132
	90	5.31	5.05	0.34	0.569
	170	4.46	4.62	0.39	0.328
A: G Ratio	0	0.68	0.94	0.06	0.425
	90	0.61	0.62	0.05	0.823
	170	0.84	0.73	0.03	0.164
AST (U/L)	0	110.32	112.58	6.35	0.432
	90	114.68	116.58	4.61	0.579
	170	115.69	117.54	7.72	0.620
ALT (U/L)	0	66.23	75.48	8.97	0.720
	90	67.58	72.59	7.58	0.354
	170	70.61	76.28	6.59	0.679
Urea (mg/dL)	0	27.86	29.74	1.95	0.450
	90	33.23	32.32	1.74	0.112
	170	36.56 ^b^	32.71 ^a^	1.68	0.042

^a,b^ Mean values bearing different superscripts within a row vary significantly (*p* < 0.05).

**Table 4:** Antioxidants status of buffalo calves fed a diet containing a garlic oil-cinnamon bark blend.

Attributes	Treatments		SEM	*p* Value
	CONT (n = 8)	GOCB (n = 8)		
T-SH *(*μmol mg^−1^ Hb)	188.31 ^a^	285.47 ^b^	35.48	0.006
GSH (μmol mg^−1^ Hb)	14.25 ^a^	28.49 ^b^	2.57	0.002
Catalase (mmol mg^−1^ Hb)	1.01 ^a^	6.12 ^b^	0.39	0.003
SOD (mmol MTT formazon formed mg^−1^ Hb)	0.13 ^a^	0.28 ^b^	0.04	0.006
LPO (nmol MDA mg^−1^ Hb)	10.28 ^b^	7.6 ^a^	1.56	0.009

^a,b^ Mean values with different superscripts within a row differ significantly (*p* < 0.01).

**Table 5:** Effect of garlic oil-cinnamon bark blend supplementation on delayed type of hypersensitivity (DTH) reaction (%) to phyto-haemagglutinin-P in buffaloes.

Hours (Post-Injection)	Treatments		SEM	*p* Value	Period Mean ± SE
	CONT (n = 8)	GOCB (n = 8)			
0	100	100	-	1.00	100 ^A^
24	125.71 ^a^	135.38 ^b^	8.68	0.034	130.54 ^C^ ± 2.59
48	127.08 ^a^	136.09 ^b^	10.59	0.042	131.59 ^C^ ± 3.65
72	114.94 ^a^	123.49 ^b^	5.38	0.039	119.2 ^B^ ± 2.12
96	107.41 ^a^	116.17 ^b^	2.95	0.012	111.79 ^B^ ± 2.13
Treatment mean ± SE	115.03 ^a^ ± 1.93	122.23 ^b^ ± 3.26	10.56	0.024	

Mean values bearing ^a,b/A,B,C^ superscripts within a row/column vary significantly (*p* < 0.05).

**Table 6:** Effect of garlic oil-cinnamon bark supplementation on antibody titre (log10) against *Pasteurella multocida* in buffaloes.

Days Post-Immunisation	Antibody Titre (log10)		SEM	*p* Value
	CONT (n = 8)	GOCB (n = 8)		
0	1.76	1.72	0.05	0.322
30	1.86 ^a^	1.98 ^b^	0.06	0.019
60	1.88 ^a^	2.05 ^b^	0.07	0.048

^a,b^ mean values with different superscripts within a row differ significantly (*p* < 0.05).

**Table 7:** Nutrient digestibility (%) of buffalo calves fed a feed containing a garlic oil-cinnamon bark blend.

Attributes	Treatments		SEM	*p* Value
	CONT (n = 8)	GOCB (n = 8)		
Dry Matter	56.72 ^a^	60.20 ^b^	0.874	0.036
Organic Matter	60.28 ^a^	69.28 ^b^	1.68	0.001
Crude Protein	61.58 ^a^	67.66 ^b^	1.21	0.002
Ether Extract	69.82	72.75	2.73	0.621
Neutral Detergent Fibre	47.56	50.70	1.34	0.263
Acid Detergent Fibre	43.27	48.40	1.79	0.162

^a,b^ Mean values with different superscripts within a row differ significantly (*p* < 0.05).

**Table 8:** Influence of supplementing different feed additives on in vivo methane production (methane concentration in exhaled air, ppm).

Attribute	Treatments		SEM	*p* Value
	CONT (n = 8)	GOCB (n = 8)		
Methane conc.	431.29 ^b^	285.19 ^a^	27.16	0.004

^a,b^ mean values with different superscripts within a row varies significantly (*p* < 0.01).

## Data Availability

The original contributions presented in this study are included in the article. Further inquiries can be directed to the corresponding authors.

## References

[1] Paul S.S., Dey A. (2015). Nutrition in health and immune function of ruminants. Indian J. Anim. Sci..

[2] Niu X., Ding Y., Chen S., Gooneratne R., Ju X. (2022). Effect of immune stress on growth performance and immune functions of livestock: Mechanisms and prevention. Animals.

[3] Klasing K.C. (2007). Nutrition and the immune system. Br. Poult. Sci..

[4] Chen J., Li Y., Tian Y., Huang C., Li D., Zhong Q., Ma X. (2015). Interaction between microbes and host intestinal health: Modulation by dietary nutrients and gut-brain-endocrine-immune axis. Curr. Protein Pept. Sci..

[5] Pahwa H., Sharan K. (2022). Food and nutrition as modifiers of the immune system: A mechanistic overview. Trends Food Sci. Technol..

[6] Getabalew M., Alemneh T., Akeberegn D. (2019). Methane production in ruminant animals: Implication for their impact on climate change. Concepts Dairy Vet. Sci..

[7] Firkins J.L., Mackie R.I. (2020). Ruminal protein breakdown and ammonia assimilation. Improving Rumen Function.

[8] Sheoran S., Dey A., Sindhu S. (2023). Reduction of methane and nitrogen emission and improvement of feed efficiency, rumen fermentation, and milk production through strategic supplementation of eucalyptus (*Eucalyptus citriodora*) leaf meal in the diet of lactating buffalo (*Bubalus bubalis*). Environ. Sci. Pollut. Res..

[9] Kholif A.E. (2024). The Impact of Varying Levels of Laurus nobilis Leaves as a Sustainable Feed Additive on Ruminal Fermentation: In Vitro Gas Production, Methane and Carbon Dioxide Emissions, and Ruminal Degradability of a Conventional Diet for Ruminants. Fermentation.

[10] Valdivieso-Ugarte M., Gomez-Llorente C., Plaza-Díaz J., Gil Á. (2019). Antimicrobial, antioxidant, and immunomodulatory properties of essential oils: A systematic review. Nutrients.

[11] Amer S.A., Abdel-Wareth A.A.A., Gouda A., Saleh G.K., Nassar A.H., Sherief W.R.I.A., Albogami S., Shalaby S.I., Abdelazim A.M., Abomughaid M.M. (2022). Impact of dietary lavender essential oil on the growth and fatty acid profile of breast muscles, antioxidant activity, and inflammatory responses in broiler chickens. Antioxidants.

[12] Calsamiglia S., Busquet M., Cardozo P.W., Castillejos L., Ferret A. (2007). Invited review: Essential oils as modifiers of rumen microbial fermentation. J. Dairy Sci..

[13] DAHD (2019). 20th Livestock Census-2019 All India Report.

[14] ICAR (2024). Nutrient Requirement of Animals-Buffalo.

[15] Patra A.K. (2014). Trends and projected estimates of GHG emissions from Indian livestock in comparisons with GHG emissions from world and developing countries. Asian-Australas. J. Anim. Sci..

[16] Dey A., Paul S.S., Lailer P.C., Dahiya S.S. (2021). Reducing enteric methane production from buffalo (*Bubalus bubalis*) by garlic oil supplementation in in vitro rumen fermentation system. SN Appl. Sci..

[17] Sari N.F., Ray P., Rymer C., Kliem K.E., Stergiadis S. (2022). Garlic and its bioactive compounds: Implications for methane emissions and ruminant nutrition. Animals.

[18] Ali A., Ponnampalam E.N., Pushpakumara G., Cottrell J.J., Suleria H.A., Dunshea F.R. (2021). Cinnamon: A natural feed additive for poultry health and production—A review. Animals.

[19] Tedeschi L.O., Muir J.P., Naumann H.D., Norris A.B., Ramírez-Restrepo C.A., Mertens-Talcott S.U. (2021). Nutritional aspects of ecologically relevant phytochemicals in ruminant production. Front. Vet. Sci..

[20] Oh J., Hristov A.N., Lee C., Cassidy T., Heyler K., Varga G.A., Pate J., Walusimbi S., Brzezicka E., Toyokawa K. (2013). Immune and production responses of dairy cows to postruminal supplementation with phytonutrients. J. Dairy Sci..

[21] Imbabi T., Hassan T.M., Osman A., El Aziz A.H.A., Tantawi A.A., Nasr M.A. (2024). Impacts of thyme and/or garlic oils on growth, immunity, antioxidant and net farm income in Damascus goats. Sci. Rep..

[22] Chaves A.V., Dugan M.E.R., Stanford K., Gibson L.L., Bystrom J.M., McAllister T.A., Van Herk F., Benchaar C. (2011). A dose-response of cinnamaldehyde supplementation on intake, ruminal fermentation, blood metabolites, growth performance, and carcass characteristics of growing lambs. Livest. Sci..

[23] Singh R.K., Dey A., Paul S.S., Singh M., Dahiya S.S., Punia B.S. (2020). Associative effects of plant secondary metabolites in modulating in vitro methanogenesis, volatile fatty acids production and fermentation of feed in buffalo (*Bubalus bubalis*). Agrofor. Syst..

[24] Kumar K. (2017). Effects of Feed Additives Rich in Essential Oils on Rumen Fermentation, Methanogenesis and Nutrient Utilization in Buffalo. Master’s Thesis.

[25] Sedlak J., Lindsay R.H. (1968). Estimation of total, protein-bound, and nonprotein sulfhydryl groups in tissue with Ellman’s reagent. Anal. Biochem..

[26] Prins H.K., Loos J.A. (1969). Biochemical Methods in Red Cell Genetics.

[27] Bergmeyer H. (1983). UV method of catalase assay. Methods of Enzymatic Analysis.

[28] Madesh M., Balasubramanian K. (1998). Microtiter plate assay for superoxide dismutase using MTT reduction by superoxide. Indian J. Biochem. Biophys..

[29] Placer Z.A., Cushman L.L., Johnson B.C. (1966). Estimation of product of lipid peroxidation (malonyl dialdehyde) in biochemical systems. Anal. Biochem..

[30] Richateich R. (1969). Clinical Chemistry Theory and Practice.

[31] Abbas A.K., Lichtman A.H., Pillai S. (2014). Cellular and Molecular Immunology E-Book.

[32] AOAC. Association of Official Analytical Chemistry–AOAC (2007). Official Methods of Analysis.

[33] Van Soest P.J., Robertson J.B., Lewis B.A. (1991). Methods for dietary fiber, neutral detergent fiber, and nonstarch polysaccharides in relation to animal nutrition. J. Dairy Sci..

[34] (2011). SPSS.

[35] Snedecor G., Cochran W. (1994). Statistical Methods.

[36] Lawson L.D., Koch H.P., Lawson L.D. (2006). The Composition and Chemistry of Garlic Cloves and Processed Garlic.

[37] Ozma M.A., Abbasi A., Rezaee M.A., Hosseini H., Sabahi N.H.S., Noori S.M.A., Sepordeh S., Khodadadi E., Lahouty M., Kafil H.S. (2023). A critical review on the nutritional and medicinal profiles of garlic’s (*Allium sativum* L.) bioactive compounds. Food Rev. Int..

[38] Mirunalini S., Dhamodharan G., Karthishwaran K. (2010). A natural wonder drug helps to prevent cancer: Garlic oil. Not. Sci. Biol..

[39] Ding H., Ao C., Zhang X. (2023). Potential use of garlic products in ruminant feeding: A review. Anim. Nutr..

[40] Borzoei A., Rafraf M., Niromanesh S., Farzadi L., Narimani F., Doostan F. (2018). Effects of cinnamon supplementation on antioxidant status and serum lipids in women with polycystic ovary syndrome. J. Tradit. Complement. Med..

[41] Jamroz D., Wertelecki T., Houszka M., Kamel C. (2006). Influence of diet type on the inclusion of plant origin active substances on morphological and histochemical characteristics of the stomach and jejunum walls in chicken. J. Anim. Physiol. Anim. Nutr..

[42] Garcia-Gonzalez R., Lopez S., Fernandez M., Bodas R., Gonzalez J.S. (2008). Screening the activity of plants and spices for decreasing ruminal methane production in vitro. Anim. Feed. Sci. Technol..

[43] Luo Q., Li N., Zheng Z., Chen L., Mu S., Chen L., Liu Z., Yan J., Sun C. (2020). Dietary cinnamaldehyde supplementation improves the growth performance, oxidative stability, immune function, and meat quality in finishing pigs. Livest. Sci..

[44] Jakhmola R.C., Sahoo A., Tripathi M.K., Sharma T. (2011). Phytochemicals in Animal Nutrition. Proceedings of the 1st Conference of Indian Academy of Veterinary Nutrition and Animal Welfare, Chhattisgarh, India, 11–12 September 2011.

[45] Ramdani D., Yuniarti E., Jayanegara A., Chaudhry A.S. (2023). Roles of essential oils, polyphenols, and saponins of medicinal plants as natural additives and anthelmintics in ruminant diets: A systematic review. Animals.

[46] Wang F., Xu R., Zheng F., Liu H. (2018). Effects of triclosan on acute toxicity, genetic toxicity and oxidative stress in goldfish (*Carassius auratus*). Exp. Anim. Tokyo.

[47] Venskutonis P.R., Gruzdien A., Tirzite D., Tirzitis G. (2005). Assessment of antioxidant activity of plant extracts by different methods. Acta Hortic..

[48] Droge W. (2002). Aging-related changes in the thiol/disulfide redox state: Implications for the use of thiol antioxidants. Exp. Gerontol.

[49] Nascimento L.D.D., de Moraes A.A.B., da Costa K.S., Galúcio J.M.P., Taube P.S., Costa C.M.L., Cruz J.N., de Aguiar Andrade E.H., de Faria L.J.G. (2020). Bioactive natural compounds and antioxidant activity of essential oils from spice plants: New findings and potential applications. Biomolecules.

[50] Kumar K., Dey A., Rose M.K., Dahiya S.S. (2022). Impact of dietary phytogenic composite feed additives on immune response, antioxidant status, methane production, growth performance and nutrient utilization of buffalo (*Bubalus bubalis*) calves. Antioxidants.

[51] El-Naggar S., Ibrahim E.M. (2018). Impact of incorporating garlic or cumin powder in lambs ration on nutrients digestibility, blood constituents and growth performance. Egypt. J. Nutr. Feed..

[52] Lee J.S., Kang S., Kim M.J., Han S.G., Lee H.G. (2020). Dietary supplementation with combined extracts from garlic (Allium sativum), brown seaweed (*Undaria pinnatifida*), and pinecone (*Pinus koraiensis*) improves milk production in Holstein cows under heat stress conditions. Asian-Australas. J. Anim. Sci..

[53] Kewan K.Z., Ali M.M., Ahmed B.M., El-Kolty S.A., Nayel U.A. (2021). The effect of yeast (*Saccharomyces cerevisiae*), garlic (*Allium sativum*) and their combination as feed additives in finishing diets on the performance, ruminal fermentation, and immune status of lambs. Egypt. J. Nutr. Feed..

[54] El-Azrak K.E.D.M., Morsy A.S., Soltan Y.A., Hashem N.M., Sallam S.M.A. (2022). Impact of specific essential oils blend on milk production, serum biochemical parameters and kid performance of goats. Anim. Biotechnol..

[55] Cho J.H., Chen Y., Min B.J., Kim H.J., Kwon O.S., Shon K.S., Kim I.-S., Kim S.J., Asamer A. (2006). Effects of essential oils supplementation on growth performance, IgG concentration and fecal noxious gas concentration of weaned pigs. Asian-Aust. J. Anim. Sci..

[56] Mir P.S., Mears G.J., Okine E.K., Entz T., Ross C.M., Husar S.D., Mir Z. (2000). Effects of increasing dietary grain on viscosity of duodenal digesta and plasma hormone, glucose and amino acid concentrations in steers. Can. J. Anim. Sci..

[57] Khiaosa-Ard R., Zebeli Q. (2013). Meta-analysis of the effects of essential oils and their bioactive compounds on rumen fermentation characteristics and feed efficiency in ruminants. J. Anim. Sci..

[58] Kholif A.E., Olafadehan O.A. (2021). Essential oils and phytogenic feed additives in ruminant diet: Chemistry, ruminal microbiota and fermentation, feed utilization and productive performance. Phytochem. Rev..

[59] Ma T., Chen D., Tu Y., Zhang N., Si B., Deng K., Diao Q. (2016). Effect of supplementation of allicin on methanogenesis and ruminal microbial flora in Dorper crossbred ewes. J. Anim. Sci. Biotechnol..

[60] El-Essawy A.M., Abdou A.R., El-Gendy M.H. (2019). Impact of Anise, Clove, and Thyme essential oils as feed supplements on the productive performance and digestion of Barki ewes. Aust. J. Basic Appl. Sci..

[61] Wallace R.J., McEwan N.R., McIntosh F.M., Teferedegne B., Newbold C.J. (2002). Natural products as manipulators of rumen fermentation. Asian-Aust. J. Anim. Sci..

[62] Walker N.D., Newbold C.J., Wallace R.J. (2005). Nitrogen metabolism in the rumen. Nitrogen and Phosphorus Nutrition of Cattle: Reducing the Environmental Impact of Cattle Operations.

[63] Lambo M.T., Ma H., Liu R., Dai B., Zhang Y., Li Y. (2024). Mechanism, effectiveness, and the prospects of medicinal plants and their bioactive compounds in lowering ruminants’ enteric methane emission. Animal.

[64] Honan M., Feng X., Tricarico J.M., Kebreab E. (2021). Feed additives as a strategic approach to reduce enteric methane production in cattle: Modes of action, effectiveness and safety. Anim. Prod. Sci..

[65] Mbiriri D., Cho S., Mamvura C., Choi N. (2015). Assessment of rumen microbial adaptation to garlic oil, carvacrol and thymol using the consecutive batch culture system. J. Vet. Sci. Anim. Husb..

[66] Patra A.K., Yu Z. (2015). Effects of garlic oil, nitrate, saponin and their combinations supplemented to different substrates on in vitro fermentation, ruminal methanogenesis, and abundance and diversity of microbial populations. J. Appl. Microbiol..

